# Emerging SARS-CoV-2 Variants: A Review of Its Mutations, Its Implications and Vaccine Efficacy

**DOI:** 10.3390/vaccines9101195

**Published:** 2021-10-18

**Authors:** Sindhu Ramesh, Manoj Govindarajulu, Rachel S. Parise, Logan Neel, Tharanath Shankar, Shriya Patel, Payton Lowery, Forrest Smith, Muralikrishnan Dhanasekaran, Timothy Moore

**Affiliations:** 1 Department of Drug Discovery and Development, Auburn University Harrison School of Pharmacy, Auburn, AL 36849, USA; szr0065@auburn.edu (S.R.); myg0003@auburn.edu (M.G.); res0071@auburn.edu (R.S.P.); ltn0005@auburn.edu (L.N.); pll0007@auburn.edu (P.L.); smithft@auburn.edu (F.S.); dhanamu@auburn.edu (M.D.); 2 Department of Internal Medicine, Ramaiah Medical College and Hospital, Bengaluru 560054, Karnataka, India; tharanath37@msrmc.ac.in; 3 Department of Neuroscience, Middlebury College, Middlebury, VT 05753, USA; shriyap@middlebury.edu

**Keywords:** SARS-CoV-2, COVID-19, variants of concern, variants of interest, mutations, vaccines

## Abstract

The widespread increase in multiple severe acute respiratory syndrome coronavirus-2 (SARS-CoV-2) variants is causing a significant health concern in the United States and worldwide. These variants exhibit increased transmissibility, cause more severe disease, exhibit evasive immune properties, impair neutralization by antibodies from vaccinated individuals or convalescence sera, and reinfection. The Centers for Disease Control and Prevention (CDC) has classified SARS-CoV-2 variants into variants of interest, variants of concern, and variants of high consequence. Currently, four variants of concern (B.1.1.7, B.1.351, P.1, and B.1.617.2) and several variants of interests (B.1.526, B.1.525, and P.2) are characterized and are essential for close monitoring. In this review, we discuss the different SARS-CoV-2 variants, emphasizing variants of concern circulating the world and highlight the various mutations and how these mutations affect the characteristics of the virus. In addition, we discuss the most common vaccines and the various studies concerning the efficacy of these vaccines against different variants of concern.

## 1. Introduction

The commonly known severe acute respiratory syndrome coronavirus 2 (SARS-CoV-2) is responsible for the coronavirus disease 2019 (COVID-19) that initially appeared by public reports in December 2019 in China and then spread worldwide, causing a pandemic outbreak throughout 2020 and 2021. As of 24th September 2021, COVID-19 has led to approximately 230 million confirmed cases and caused over 4.7 million deaths worldwide. In the United States, there have been around 42 million confirmed cases of COVID-19, with 677,323 deaths [1].

Viral variants result from mutations during viral replication. A mutation is described as any change, such as a substitution, deletion, or addition, in a genetic sequence of a virus compared to the normal sequence. Coronaviruses are positive, single-stranded RNA viruses resembling a crown appearance under a microscope [2]. The mutation rate is slow compared to other common viruses, such as influenza [3]. This means SARS-CoV-2 is less likely to experience mutational changes, such as antigenic drift and antigenic shift responsible for altering the virus composition that leads to differences in infectivity, transmission, and disease severity. As COVID-19 spreads across the world, the virus naturally mutates to form new variants that can either be more or less infectious than the previous form depending on the altered composition. Some of the mutations, especially those occurring at the Spike (S) protein, could affect the entry of the virus into the target cells and the efficacy of the antibody protection. Specifically, mutations occurring in the Receptor-Binding Domain (RBD) of the S protein are of high significance as most vaccines and neutralizing antibodies target the RBD [4]. Other mutations in the S protein, such as one occurring at the N-terminal Domain (NTD), could impair the capability of the neutralizing antibodies as well [5]. With more studies, the impact of mutations occurring in other regions of the genome will be determined. 

The D614G mutation in the S protein documented in the early part of the pandemic is found in almost every sequence worldwide. This mutation is characterized by the replacement of aspartic acid with glycine at position 614 of the S protein and influences viral infectivity [6]. Higher levels of viral RNA were noted in the patients, indicating high viral load and potential for higher infectivity [7]. As the transmission of the virus continued, several new variants with multiple mutations have emerged globally [8]. The Center for Disease Control (CDC), in collaboration with SARS-CoV-2 Interagency Group (SIG), classify SARS-CoV-2 variants into variants of concern (VOC), variant of interest (VOI), and variants of high consequence depending on the threat level they pose to the public’s health, as described in the following sections [9]. 

In this review, the various prevalent SARS-CoV-2 variants are identified to determine their impact on altering the current disease pathology. The focus is on the variants of concern, its multiple mutations, and their consequences. Furthermore, we compare the different COVID-19 variants to understand the underlying changes and how they could impact the current pandemic, at-risk patient populations, and healthcare professionals and facilities if certain variants become more emergent than the original strain. 

## 2. Nomenclature of SARS-CoV-2 Variants

The naming of the SARS-CoV-2 variants is based on Phylogenetic Assignment of Named Global Outbreak Lineages (PANGOLIN) or interchangeably referred to as Pango lineage nomenclature [10]. According to the nomenclature, there are two major lineages, namely, A and B, at the root of the phylogeny of SARS-CoV-2. Lineage A viruses, for instance, the Wuhan/WHO4/2020 sequence sampled in January 2020, share two nucleotides (positions 8782 in ORF1ab and 28144 in ORF8) with the closest known bat viruses (RaTG13 and RmYN02). In comparison, lineage B, such as the Wuhan-Hu-1 strain sampled in December 2019, display different nucleotides at the above-mentioned sites. The additional SAR-CoV-2 genomes, which descend from either lineage A or lineage B, are designated a numerical value, for example, lineage A.1 or lineage B.2. Furthermore, these lineages (A.1 or lineage B.2) can act as predecessors for virus lineages that emerge in other geographical areas or at different time points, and these are designated with two sublevels, for instance, A.1.1. These designations can proceed for a maximum of three sublevels (e.g., A.1.1.1), after which new descendent lineages are given a letter (in English alphabetical sequence from C, so A.1.1.1.1 would become C.1 and A.1.1.1.2 would become C.2). These descendent lineages should show phylogenetic evidence of emergence from an ancestral lineage into another geographically distinct population, implying substantial forward transmission in that population. The following criteria are used to determine phylogenetic evidence for a new lineage:

- Exhibits one or more shared nucleotide differences from the ancestral lineage
- Comprises at least five genomes with >95% of the genome sequenced
- Exhibits at least one shared nucleotide change among genomes within the lineage
- Demonstrate a bootstrap value >70% for the lineage-defining node.

As of September 2021, lineage B and its sub-lineage B.1 appears to be the most prevalent worldwide. In the United States, there are four circulating variants, with B.1.1.7 being the most common. The other variants include B.1.351, P.1 (B.1.1.28.1), and B.1.617.2 (the B.1.427 and B.1.429 variants have been de-escalated due to low prevalence). These variants appear to spread more easily and quickly than other variants, leading to more cases of COVID-19. The CDC categorizes SARS-CoV-2 variants into different groups regarding the potential for causing severe disease leading to morbidity and/or mortality, significant infectivity rate, or decreased response to SARS-CoV-2 antibodies generated from a previous infection or vaccination [11]. The different variants have been categorized into variants of interest (VOI), variants of concern (VOC), or variants of high consequence (VOHC) in the United States (Table 1). Conversely, the European Centre for Disease Prevention and Control categorizes them into variants of interest (VOI), variants of concern (VOC), or variants under monitoring (VOM). 

The World Health Organization (WHO) revised the naming system for SARS-CoV-2 (both VOC and VOI) based on the Greek alphabet, such as “Alpha”, “Beta” or “Gamma”. This system has the advantage of referring to the variants more quickly in a simplified scientific language, especially for non-scientists, national authorities, media, and others. Additionally, it avoids identifying them by the countries where they were identified first; thereby, preventing stigmatization of a country for detecting and reporting variants. The variants using the new WHO nomenclature are found in Table 1. 

## 3. SARS-CoV-2 Genome

**Figure 1:**
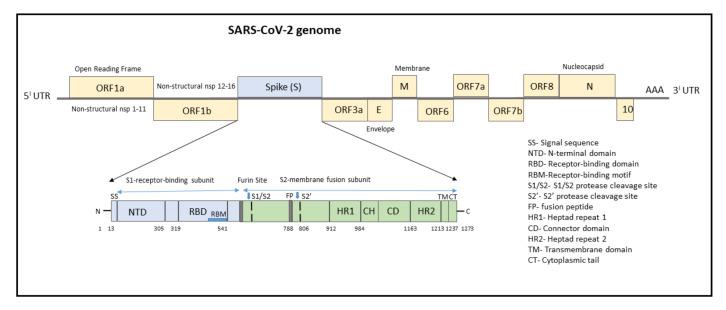
Structure of SARS-CoV-2 genome with domain structure of the Spike protein.

The genome of SARS-CoV-2 consists of a single-stranded positive-sense RNA consisting of 5′-UTR (untranslated region) and a poly(A)-tail at 3′-UTR, both of which assumes a structure similar to the mRNA of host cells (Figure 1). The proteins of SARS-CoV-2 consist of two large polyproteins: ORF1a and ORF1ab (that proteolytically cleave to form 16 nonstructural proteins), major transmembrane Spike (S) glycoprotein, membrane (M), nucleocapsid (N) protein, and small envelope (E) protein, and at least six accessory proteins: ORF3a, ORF6, ORF7a, ORF7b, ORF8a, and ORF8b, which follows a typical 5′-3′ order of appearance [12]. 

The ORF1a/b encodes a polyprotein termed polyprotein1a (pp1a), which corresponds to nonstructural proteins NSP1 to NSP11, and pp1b consisting of NSP12 to NSP16. Several functional domains of NSPs have been studied, notably the 3C-like cysteine proteinase (3CL^pro^, nsp5), RNA-dependent RNA polymerase (RdRp, nsp12), nidovirus RdRp-associated nucleotidyltransferase (N terminal of nsp12), helicase (Hel, nsp13), and exonuclease (ExoN, nsp14) [12,13]. Other nsps have a role in suppressing host cells, immunological suppression, and other functions. ORF1a Nsps are critical for controlling genome expression, while ORF1b Nsps are important for replication. 

The structural RNA genome transcribes into the S protein with a size of 180–200 kDa, which plays a role in virus entry into host cells by binding to the human angiotensin-converting enzyme 2 (ACE2) receptor. The S protein consists of 1273 amino acids and consists of a signal sequence (aa 1–13), S1 subunit (14–685 residues), and S2 subunit (686–1273 residues), as shown in Figure 1. 

The signal sequence consisting of 13 aa has high hydrophobic residues and helps in guiding the S protein to its membrane destination. The S1 subunit consists of the N-terminal domain (14–305) and receptor-binding domain (319–541). The N-terminal domain (NTD) plays a role in attachment, and mutations at NTD confer reduced sensitivity to neutralizing antibodies, which makes SARS-CoV-2 more permissive for deleterious escape mutations in the RBD. Neutralizing antibodies from both infected patients and vaccinated individuals target RBD and NTD. For instance, NTD-targeting antibodies bind to NTD to form an NTD/antibody complex, thereby preventing conformational changes in the S protein and block membrane fusion and viral entry [14]. The RBD plays a critical role in binding to the host cell ACE2 receptor in the region of aminopeptidase N. Studies indicate that the SARS-CoV-2 RBM has more residues that directly interact with the ACE2 receptor than the SARS-RBD [15]. Hence, mutations in the key residues in this region play an important role in enhancing the interaction with ACE2. The RBD functions to interact with human angiotensin-converting enzyme II (ACE2) and facilitates virus entry into host cells. The RBD-targeting neutralizing antibodies bind directly to S Protein RBD and compete for the ACE2 receptor resulting in neutralization of the virus [14]. 

The S2 subunit, which contributes to membrane fusion, consists of the fusion peptide (788–806), heptapeptide repeat sequence 1 (912–984), HR2 (1163–1213), TM domain (1213–1237), and cytoplasm domain (1237–1273). The furin-like cleavage domain is the site at which S protein gets cleaved into two parts, the S1 subunit and S2 subunit. SARS-CoV-2 possesses multiple furin cleavage sites, thereby increasing the probability of being cleaved by the host furin-like proteases. This induced cell–cell fusion to form syncytium to facilitate viral spread from one cell to another, thereby increases the chances of infectivity [16]. An additional cleavage site, referred to as the S2′site, is cleaved by the host TMPRSS2, where S2 is cleaved into FP and S2′domains. Hence, furin cleavage plays an important role in viral assembly, whereas TMPRSS2 cleavage triggers membrane fusion, syncytium formation, and viral entry into a target cell [17]. 

After the S protein gets cleaved, the fusion peptide (FP) undergoes a conformational change and inserts into the host membrane and anchors inside. The TM and CD stabilize the trimeric structure during the process of viral fusion and forms an anchor inside the virion [18]. Additionally, FP has been shown to mediate membrane fusion by disrupting and linking lipid bilayers of the host cell membrane [19]. Once the distance between the viral and host membrane is shortened, the HR1 and HR2 undergo conformational changes and mediates viral fusion and entry of the S2 subunit by bringing the viral envelope and host cell membrane close to one another [20]. 

Since the S protein is the main antigenic component of SARS-CoV-2, neutralizing antibodies targeting the S protein can induce protective immunity against the infection. Antibodies targeting various regions of the S protein have different mechanisms in preventing SARS-CoV-2 infection. For instance, monoclonal antibodies (mAbs) and nanobodies (Nbs) targeting the RBD form RBD/mAb or RBD/Nb complexes, which inhibit the binding of the RBD to ACE2, thereby prevent the entry of SARS-CoV-2 into the host cells. Conversely, mAbs targeting the NTD form the NTD/mAb complex and prevent conformational changes in the S protein and block membrane fusion and viral entry. However, RBD-targeting antibodies are more potent than the antibodies targeting other regions (such as NTD) of the S protein, but they might exhibit reduced efficacy in inhibiting multiple virus strains [14].

The envelope (E) protein, being the smallest structural protein, with a molecular weight of 8–12 kDa, possesses three important domains viz. (N)-terminus, transmembrane domain (TMD), and (C)-terminus. The (C)-terminal domain (motif DLLV) binds to human PALS1, a tight junction-associated protein, which is essential for the establishment and maintenance of epithelial polarity in mammals [21]. The E protein is involved in pathogenesis, virus assembly, and release [22]. Additionally, in cooperation with the M protein, it mediates host immune responses by the activation of NLRP3 inflammasome and PDZ binding function via its C-terminal domain. 

The membrane (M) protein is a 25–30 kDa O-linked glycoprotein, binds to structural proteins, such as nucleocapsid, and promotes viral assembly, and increases its virulence [23]. The nucleocapsid (N) protein is responsible for RNA packaging, virus particle releasing, and the ribonucleoprotein core forming process. Mutations in the N protein pose a potential diagnostic risk as most commercially available antigenic rapid diagnostic tests detect the presence of the N protein [24]. 

The ORF3a located between the S and E genes is important for regulating apoptosis and inflammatory responses in the infected cells [25,26]. Additionally, ORF3a activates the innate immune signaling receptor NLRP3 inflammasome and causes tissue inflammation and cytokine production [27]. The ORF6 is important for increasing the viral infectivity by suppressing interferon production and interferon signaling [28]. The ORF7a and ORF7b are transmembrane proteins important for structural integrity, while the ORF8 of SARS-CoV-2 mediates the immune evasion by interacting with major histocompatibility complex molecules class I (MHC-I) and down-regulating the surface expression of MHC-I in various cells [29]. ORF9b associates with an adaptor protein, TOM70, and thereby suppresses IFN-I-mediated antiviral responses [30]. The SARS-CoV-2 ORF10 protein interacts with multiple human proteins expressed in the lung tissues and is found to maintain disease transmissibility [31]. Additionally, apart from viral replication, the above-mentioned accessory proteins play a role in host immune escape [32,33]. 

## 4. Mutations Associated with SARS-CoV-2 Genome

Some of the most important mutations can influence features and may modify infectivity, disease severity, or interactions with host immunity. However, the evolution of SARS-CoV-2 has been remarkable because of the development of mutations in terms of “variants of concern” which affect virus features, such as transmissibility and antigenicity, most likely in reaction to the altering immunological profile of the human population. There is accumulating data that postvaccination serum diminishes the neutralization of some SARS-CoV-2 variants; nevertheless, a better knowledge of correlations of safety is needed to assess how this may possibly influence vaccine effectiveness. The following section discusses the most important mutations in different regions of the SARS-CoV-2 genome with functional consequences. 

Mutations on 5′UTR: The 5′untranslated region (5′UTR) plays a critical role in the regulation of translation and the gene expression of the virus [34]. The most common mutations include Mutation C241T, A187G, G199T, C222T, G208T, C218T, G242T, and A169G. The C241T mutation does not alter the protein sequence; however, it might have effects on the secondary RNA structure and, therefore, influence the rate of RNA replication and viral infection cycle [35]. 

Mutations on ORF1ab: The ORF1ab consists of several NSPs that play an important role in viral RNA synthesis, and the most common and significant mutations in the NSPs are discussed. 

NSP1: The major functions of NSP1 include viral replication and inactivation of the type-1 interferon-induced antiviral system [36]. One important mutation, the deletion of three amino acids (KSF) at the AA position 241–243, has been shown to affect the structure of the C-terminal region of the protein, which is important for the regulation of viral replication and negative effect on the host’s gene expression [37]. The deletion at AA 500–532 is associated with retained ribosomal binding ability while affecting mRNA metabolic process, higher RT-PCR cycle thresholds, and lower serum IFN-β levels of infected patients [38].

NSP2: The T265I mutation in NSP2 is commonly associated with D614G substitution of the S protein, and the co-occurrence of these mutations could have direct structural interactions conferring protein stability or regulating other proteins important for viral immune evasion [39]. The other mutation is T85I, an infectivity-strengthening mutation, which may mainly benefit from the co-mutation with other infectivity-strengthening mutations, such as D614G and Q57H.

NSP3: The main function of NSP3 is the formation and activity of the viral replication/transcription complex. It is also involved in inflammation as it interacts with the inflammasome complex. Mutation F206F affects the fitness of the virus and is a part of G glade (four mutations—NSP12, 5′ UTR, D614G of Spike, and NSP3) that originated in China [40]. The S1197R and T1198K mutations in the NSP3 protein have been related to increased severity of infection [41]. 

NSP12: NSP12 plays a crucial role in SARS-CoV-2 replication and transcription machinery [42]. Mutation P323L is a high-frequency mutation, does not alter the functionality of NSP12 but co-mutates with T85I, D614G, Q57H, and S24L simultaneously. Specifically, mutations with D614G enhance the mutation of D614G, thereby enhancing the transmission capability of SARS-CoV-2 [43]. 

NSP13: NSP13 belongs to superfamily 1 helicase, which unwinds a double-stranded RNA (dsRNA) or DNA (dsDNA) in the 5′ to 3′ direction into two single-stranded nucleic acids [28]. Two mutations, Y541C and P504L, are important and help to destabilize the structure of NSP13, inhibit the NSP13 from participation in the replication/transcription process, and weaken the transmission capacity of SARS-CoV-2 [27].

Mutations on ORF3a protein: The ORF3a protein is widely expressed in intracellular and plasma membranes, which induces apoptosis and inflammatory responses in the infected cells [25]. Specifically, ORF3a activates the innate immune signaling receptor NLRP3 inflammasome and causes tissue inflammation and cytokine production [27]. The Q57H mutation makes the ORF3a unstable (rigid and less flexible) and difficult to involve in the apoptosis and inflammatory response, thereby increasing the viral load in the host cell. The mutation *G251V* is also a prevalent mutation in the ORF3a protein [44].

Mutations on the ORF8 protein: The ORF8 protein of SARS-CoV-2 mediates immune evasion by interacting with major histocompatibility complex molecules class I (MHC-I) and down-regulating the surface expression of MHC-I in various cells [29]. The ORF8 protein has two high-frequency mutations, L84S and S24L. S24L may enhance SARS-CoV-2′s ability to spread by inhibiting MHC-1. Both L84S and S24L mutations make the ORF8 less rigid and more unstable, thereby evading MHC-1 and increasing the transmission [44].

Mutations on the nucleocapsid (N) protein: The nucleocapsid (N) protein is responsible for RNA packaging, virus particle releasing, and the ribonucleoprotein core forming process [45]. The high-frequency mutations detected on the nucleocapsid (N) protein are 28881G > A, 28882G > A, and 28883G > C. R203K is caused by both 28881G > A and 28882G > A. On the protein level, mutation 28883G > C leads to G204R. The mutation R203K may not affect N protein function. Mutation 28883G > C-(G204R) may affect the hydrophilicity of the N protein.

Mutations on the membrane (M) protein: The M gene encodes the most abundant viral structural protein and is responsible for the initial attachment to the host cell, assembly of viral protein, and glucose transport. Mutations in the M gene are relatively uncommon; however, there has been an increased frequency of M gene mutation, especially with the emergence of a new sub-B.1 clade, B.1.I82T. The two most important mutations include M:I82T and M:V70L in the transmembrane helices. These mutations have been shown to exhibit higher transmissibility among younger populations, and that mutations emerging in the viral membrane protein-encoding gene play crucial roles in regulating glucose uptake during viral replication. The other mutations likely to make changes in protein secondary structure include C64F, A69S, A69V, V70F, N113B, R158L, V170I, D190N, D209Y, and S214I. Alternatively, six mutations (N113B, P123L, P132S, H155Y, D190N, and T208I0) have the solvent accessibility change potential. The N113B and D190N mutations have the potential to change both protein structure and solvent accessibility in gene M [46]. 

Mutations on the envelope (E) protein: The four most common mutations in the NTD V5F, E8D, V5I, and Y2H have been detected, which might affect the viral efficiency [47]. Mutations in the TMD include T9I, F20L, L21F, V24M, and T30I and affect the homo pentameric configuration of the E protein. Mutations in the CTD, namely, S55F, V62F, R69I, and C-terminal end (DLLV: 72–75), alter the binding of the E protein to PALS1, and thus, could play a key role in COVID-19 pathogenesis. Increased mutations in the E protein of SARS-CoV-2 suggest the need for continuous monitoring of genome sequences [48]. 

Mutations on the Spike (S) protein: As the virus replicates, several mutations occur in the S protein. Mutations occurring in the RBD of the S protein affect receptor or antibody binding. Some of the most critical mutations in the S protein are depicted in Table 2. 

## 5. SARS-CoV-2 Variants of Concern

**Figure 2:**
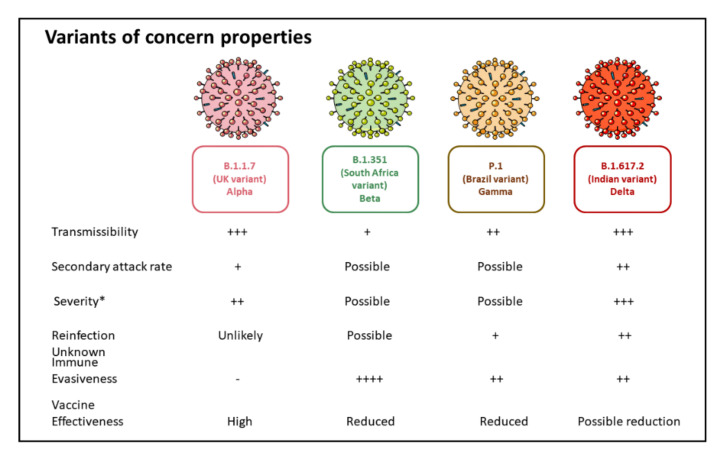
Properties of Current Variants of Concern. * Severity is determined by an increased risk of hospitalization and increased risk of mortality.

The Centers for Disease Control and Prevention (CDC) defines a variant of concern as a variant showing increased transmissibility, more severe disease, a significant reduction in neutralization by antibodies gained from previous infection or vaccination, decreased efficacy of treatments and/or vaccines, or failure in detecting by diagnostics (Figure 2). The WHO and European Centre for Disease Prevention and Control state similar definitions for reported VOC. The viruses have the inherent quality to mutate naturally over time during the process of replication. A mutation indicates a change in the genetic sequence of a virus. Hence, a “variant” means a virus strain with a collection of mutations. The variants of concern share one specific mutation termed D614G. Various studies suggest that variants with this mutation spread more quickly than viruses without this mutation [62].

### 5.1. B.1.1.7 Variant

The B.1.1.7 variant, also known as Alpha, VOC 202012/01, or 20B/501Y.V1, was first reported as a new SARS-CoV-2 variant by the United Kingdom in December 2020 [63,64]. Since then, this variant has become predominant and replaced the previous circulating viruses, especially in the United States. As previously mentioned, this variant is categorized into VOC. Recently, the WHO labeled this variant as Alpha [65]. The B.1.1.7 variant is characterized by increased transmissibility, hospitalizations, mortality rates, and burden to the health care systems [66,67]. Furthermore, there is 56% faster transmission than other lineages resulting in increased nasopharyngeal viral loads than the wild-type strain [68]. An estimate of around 35% increased risk of death has been associated with the B.1.1.7 variant compared to the pre-existing lineages, indicating the severity of the illness caused by this variant [69]. A matched cohort study from Ontario, Canada, showed increased secondary attack rates in households for B.1.1.7 index cases (1.31 times higher) in comparison to non-variant index cases [70].

**Figure 3:**
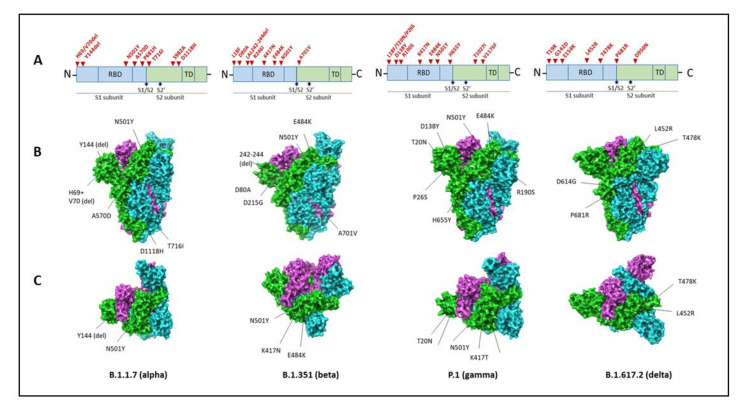
Spike mutations in the variants of concern. Amino acid substitutions and their locations in the Spike protein (**A**). The following PDB files are used for structural illustrations: 7LWV for B.1.1.7 (Alpha); 7LYN for B.1.351 (Beta); 7LWW for B.1.1.28.1 P.1 (Gamma); and 6ZGE for B.1.617.2 (Delta). Chain A: green, Chain B: blue, Chain C: purple. (**B**) Side view and (**C**) top view. The figures were prepared using Schrodinger software, and PDB files were obtained from protein databank (https://www.rcsb.org/, accessed on 9 September 2021).

The B.1.1.7 variant has 23 mutations, of which 14 are non-synonymous, 6 are synonymous, and 3 are deletions (Table 3). The N501Y, P681H, and H69-V70del mutations are the most important, as it affects the biological properties of the virus (Figure 3).

The N501Y mutation is characterized by replacing amino acid asparagine (N) with tyrosine (Y). This is concerning because it is located in the receptor-binding domain of the S protein and has been shown to increase binding affinity towards the human ACE2 receptor for viral entry. This increased binding affinity of SARS-CoV-2 to the ACE2 receptor causes increased infection and transmission, as evidenced by a retrospective study by Golubchik et al. that reported three-fold high viral titers in a group of U.K. individuals carrying the N501Y mutation [71,72]. Similarly, the N501Y mutation is associated with increased infectivity and virulence in mouse and ferret models [73]. 

The P681H mutation is located adjacent to the amino acids 682–685, the furin cleavage site (FCS) identified at the S1/S2 in the S protein. The S1/S2 furin cleavage site facilitates the viral entry into the respiratory epithelial cells and transmissibility, enhances the transmembrane serine protease (TMPRSS)-induced cleavage ability and viral infectivity [16,74]. 

The H69/V70 deletion is characterized by the loss of two amino acids (H at 69 and Valine at 70). This recurrent mutation in the amino-terminal domain of the S protein is found in at least six lineages of the SARS-CoV-2 virus in Europe. The deletion is found in over 6,000 sequences across the world and frequently occurs in combination with RBD amino acid replacements, N501Y, N439K, and Y453F. The H69/V70 deletion alters the conformation of the exposed NTD loop and is thereby associated with increased infectivity [56]. Protein structure modeling has shown the mutation modifies immunodominant epitopes in the loops of the NTD, resulting in reduced neutralization by sera from convalescent plasma and those who are vaccinated [57]. The immune escape noted with H69/V70 deletion is also contributed by Y144 deletion. The N439K has been shown to enhance the binding affinity for the ACE2 receptor and decrease the neutralizing capacity of monoclonal antibodies and convalescent sera [75].

L18F mutation in this variant alters the shape of the S protein and may help the virus evade some antibodies. Hence, this variant is associated with immune escape from neutralizing antibodies against the N-terminal domain. There is no evidence for decreased neutralization from convalescent plasma or vaccine sera, increased reinfection rates, or reduced efficacy of currently available vaccines (Figure 2) [76]. Recently, the emergence of the E484K mutation in the B.1.1.7 variant (VOC202102/02) is a concern, though no relevant data is currently available. However, the presence of E484K in other variants has been linked to reduced vaccine efficacy and immune escape from monoclonal antibodies. With respect to immune responses, antibodies generated post-B. 1.1.7 infection demonstrates poor cross-reactivity against other strains, indicating asymmetric heterotypic immunity [77]. 

The B.1.1.7 variant has demonstrated rapid transmission across the U.K., suggesting that it could become the dominant lineage and will be held responsible for future infections in Europe. This evidence is essential to monitor global surveillance, tracking continuously, and vaccine administration rates to manage the SARS-CoV-2 outbreak [78]. 

### 5.2. B.1.351 (20H/501Y.V2) Variant

The B.1.351 variant, also recognized as Beta or 20H/501Y.V2, was initially identified in December 2020 in South Africa and became the dominant circulating variant in the region. This variant was shown to have higher transmission rates. However, there has been limited evidence to indicate increased virulence or disease severity. 

The B.1.351 variant has 12 non-synonymous mutations and one deletion, of which 77% of these mutations are located in the S protein. In contrast, the remaining ones are situated in ORF1a, envelope (E), and N viral proteins [72], as illustrated in Table 3 and (Figure 3). This variant could have emerged through intra-host evolution where the virus was undergoing prolonged replication within many different individuals infected with COVID-19. This evolution is similar to the B.1.1.7 lineage. Other data supports that B.1.351 was derived as an escape variant to neutralization because multiple mutations exist in the N-terminal domain (NTD) and receptor-binding domains (RBD) of the S protein, where these regions are immunodominant [79]. Therefore, the placement of these mutations within these regions is more concerning with the B.1.351 variant regarding vaccine efficacy and treatment versus the B.1.1.7 variant. These mutations offer a selective advantage to exhibit increased transmissibility and risk of escaping neutralization and potentially acting as a treatment-resistant variant [80].

The N501Y mutation located in the RBD of the S protein confers an increased binding affinity for the ACE2 receptor, thereby increasing the viral transmission rate. Furthermore, two additional mutations in the RBD domain, K417N and E484K, play a crucial role in increasing the binding affinity of the virus with the receptor and immune evasion. The E484K mutation, localized within the receptor-binding motif (RBM), changes the configuration of the charge on the flexible loop region of the RBD, thereby leading to novel favorable contacts with the ACE2 receptor. 

The E484K mutation in the RBD is associated with reduced vaccine efficacy and immune escape from monoclonal antibodies [81]. Furthermore, there is a reduction in neutralizing antibody titers with convalescent plasma or vaccine sera, as depicted in Table 3. A recent study by Nelson et al. showed that the combination of N501Y, K417N, and E484K causes the highest degree of conformational alterations of S RBD when bound to human ACE2. In particular, two of the RBD sites (at positions 417 and 484) are key regions for the binding of neutralizing antibodies. This property allows the virus to confer a more effective escape to neutralization [82].

Mutations in the NTD region of the S protein (L18F, D80A, D215G, LAL 242–244 del, and R246I) are also of significance as NTD is also a favored target of antibodies isolated from convalescent patients or vaccinated individuals. Hence, both RBD and NTD mutations significantly impair the neutralization of the virus either by monoclonal antibodies or from sera derived from convalescent or vaccinated patients [50,83,84,85,86]. Hence, there is decreased efficacy of treatment (monoclonal antibodies and vaccines) and increased incidence of infection in the regions where the B.1.351 variant spreads predominantly. Interestingly there is no evidence to indicate that the B.1.351 variant alters the severity of COVID-19 infection [87,88]. 

### 5.3. P.1 (B.1.1.28.1) Variant

The P.1 variant, also known as Gamma or B.1.1.28.1, was initially first detected in Japan and was later identified in Brazil in January 2021, where it has become the dominant circulating virus [89]. A six-fold increase in hospital admissions was reported in January 2021 compared to December 2020, despite the population reaching high seroprevalence [90]. The P.1 variant belongs to the B.1.1.28 lineage. It contains 17 mutations, including three deletions, four nucleotide insertions, and four synonymous mutations, as described in Table 3 and depicted in Figure 3. As indicated, the P.1 variant exhibits 12 mutations in the S protein, which are implicated in increased transmission, severity, immune evasiveness, and reinfection (Figure 2). Similar to the B.1.351 variant, the E484K mutation present in the P.1 variant has been associated with immune evasion [91]. Furthermore, there is a reduced efficacy of neutralization of sera from vaccinated individuals and convalescent patients against the P.1 variant carrying E484K mutation. 

The N501Y mutation is found in all three VOCs previously talked about. The L18F, K417T, E484K, and D614G mutations are shared with the B1.351 variant. This combination of S mutations presents important roles in transmission, reinfection rates, and evasion of antibody-mediated immunity. 

The E484K substitution is associated with immune evasion and is shared among the P.1 and B.1.351 variants. Furthermore, the P.1 variant is resistant to neutralization by various RBD-directed monoclonal antibodies, attributed to E484K mutation [92]. This mutation is critical to monitor because studies have shown decreased vaccine-induced antibody neutralization titers against variants carrying the E484K substitution. This reduction was shown in both vaccination and convalescent sera. Therefore, this mutation places the variant at risk for treatment and vaccine resistance, putting populations at risk for higher transmissibility and infectivity rates. Although, sera-containing high anti-S IgG titers demonstrated neutralizing effects on the virus with the E484K mutation [93]. This indicates that vaccine schedules would need to be modified to produce high antibody levels to neutralize the variant and prevent the emergence of a new variant through resistance. 

The P.1 variant is also associated with reinfection, which can be attributed to limited and transient protective immunity generated by primary infection. At present, little is known about the severity of infection in P.1 variant-infected individuals. Due to the P.1 variant containing more mutations in the S protein, it is logical to assume it is more resistant than B.1.351 to neutralization by monoclonal antibodies and vaccine-elicited antibodies. Current data on vaccine efficacy is still being studied and is discussed in the following section. 

### 5.4. B.1.617 Variant (B.1.617.2 Variant)

The B.1.617 variant, also known as G/452R.V3, was first detected in India and has been become the dominant strain across India, other Southeast Asian countries, and the United Kingdom. This variant contains three sublineages, including B.1.617.1, B.1.617.2 (Delta), and B.1.617.3. Evidence indicates that this variant is highly contagious, evades immunity better than the existing variants, causes severe disease, and is more resistant to preventive measures, treatments, and vaccines. Currently, three sublineages have been reported. The B.1.617.3 was the first sublineage to be identified in October 2020 in India and is relatively uncommon compared to the other two sub-lineages, B.1.617.1 and B.1.617.2, which were first detected in December 2020. The U.K. government declared the B.1.617.2 subtype a “variant of concern” in the United Kingdom on 7th May 2021. Similarly, the World Health Organization and the CDC in the U.S. designated B.1.617.2 as a “variant of concern”.

Relevant mutations located in the spicule are E484Q, L452R, and P681R (Figure 3). These mutations increase transmissibility, but a percentage has not yet been reported. The B.1.617.2 variant of interest does not contain the E484Q mutation. This lineage has evidence of escaping immune response. The P681R mutation creates a more significant pathogenic potential because it enhances the binding affinity of the variant to the ACE2 receptor for entry into host cells. The other two mutations aid this activity as well. These mutations give the variant the power to evade the immune system. Preliminary data indicates the variant does not evade the Indian-made Covaxin vaccine based on virus inactivation [94]. 

Genomic and structural analysis indicates a total of 13 mutations, of which eight mutations are in the S protein. These mutations account for the increased transmissibility and the evading immune properties. In various animal models, this variant has been shown to cause severe disease [95]. Interestingly, the antibodies are less effective against this variant when compared to other variants [96]. For instance, antibodies obtained from vaccinated individuals, which could neutralize the virus, were found to be between 60% and 80% less potent against this variant. Similarly, antibodies from previously infected patients with SAR-CoV-2 neutralized B.1.617 were about 50% less effective when compared to previous variants [97]. Furthermore, there have been various reports of reinfection with this variant in individuals who have been vaccinated with the Covishield (Oxford-AstraZeneca) vaccine. 

The B.1.617 variant is characterized by two mutations in the S protein (E484Q and L452R) and is hence described as a double mutant. The E484Q mutation is similar to the one seen in the B.1.351 and P.1 variants, which have E484K mutation, whereas the L452R is present in the B.1.427/B.1.429 variants. The role of E484Q mutation in immune escape needs to be determined. Only limited studies have shown to have reduced neutralization by some but not all convalescent plasma from people who have had natural infection with SARS-CoV-2 using an experimental system [50]. Laboratory experiments have shown the L452R mutation having weak neutralization of the virus by some monoclonal antibodies and by convalescent plasma from individuals previously infected with COVID-19. The P681R mutation is located adjacent to the furin cleavage site of the S protein, meaning this mutation could alter processing or other changes controlled by the S protein. Evidence is currently lacking to understand viral changes resulting from a combination of mutations in the B.1.617 variant.

As stated earlier, the B.1.617.2 variant does not contain the E484Q mutation seen in other common variants. This variant is increasing in transmissibility across the world. As of 9th June 2021, there are 66 countries reporting cases with this variant and eight countries reporting cases related to this variant. In comparison, the B.1.1.7 lineage predominant in the United States can present with or without the E484K substitution. This particular mutation can increase infectivity rates, transmissibility, and confer treatment or vaccine resistance leading to a greater spread and increased diagnosis rates [96]. B.1.617.2 is currently classified as a VOC, as the cases are increasing worldwide, and this variant should be monitored and studied to determine vaccine efficacy. 

Researchers are interested in studying the B.1.617 SARS-CoV-2 variant that has been currently spreading in India rampantly. The Delta variant (B.1.617.2), first detected in India, has become one of the most problematic circulating viruses globally. Furthermore, it has been known to cause severe disease and increased risk of hospitalization. The Delta variant has become the most dominant strain in the United Kingdom, responsible for more than 60% of infections and causing surges of cases. According to the CDC, this variant accounted for more than 10% of all infections in the United States and is expected to increase shortly. This variant has an advantage over other strains due to multiple mutations making the strain more transmissible, making it the most dangerous variant. One study indicated B.1.617.2 might be up to 50% more contagious than the B.1.1.7 (U.K./Alpha) variant [98]. 

As of June 2021, the B.1.617.2 variant has displaced B.1.1.7 as the dominant variant in England. In the United States, B.1.1.7 tested positive cases have dropped from 70% in April 2021 to 42% in just 6 weeks and are no longer responsible for the majority of new cases. This is primarily due to the rapid growth of variants B.1.617.2 and P.1. Furthermore, at present, the growth rate of B.1.617.2 is higher than P.1 in the U.S. (0.61 vs. 0.22), and B.1.617.2 infection is growing faster in counties with a lower vaccination rate [99]. 

A preprint published by the National Institute of Virology (NIV) in Pune studied the detailed genomic and structural analysis of B.1.617, which led to identifying eight mutations in the virus’s S protein, during which it gains entry to cells [100]. Similarly, another preprint published on 5th May by NIV noticed that hamsters infected with B.1.617 had more severe lung inflammation than animals infected with other variants [97]. Hence, B.1.617.2 is further evidence of how SARS-CoV-2 persists to progress and evolve and how that progression and evolution continue to generate more hazardous variants than those that came before them.

Interestingly, scientists in the U.K. and India have noticed that complete vaccination is still largely effective against the Delta strain but may slightly be less effective than against other variants and even less so after only one dose [101]. Even fully vaccinated people appear to develop fewer neutralizing antibodies against the Delta strain than for different variants. Furthermore, the rapid spread of B.1.617.2 is cautioning that a third wave may already be in progress in the U.K. and India amongst individuals who stay unvaccinated [87]. Hence, further research needs to be conducted to study this variant to observe the danger it can create worldwide.

Another growing concern is that the “Delta” variant has further mutated to form the “Delta plus” or B.1.617.2.1 variant, known as the “AY.1” variant. The Delta plus variant was first detected in Europe in March 2021, and currently, the number of infections is increasing in India. Delta plus carries the S mutation K417N, one of the mutations found in the Beta variant (B.1.351), in addition to the key mutations found in the Delta variant. Initial data suggests that the Delta plus variant shows signs of resistance against the monoclonal antibody cocktail treatment. Furthermore, it could be possible that this variant could be able to bypass the immunity provided by the previous infection and by the vaccine. At present, the Delta plus variant is classified under a variant of concern by the Indian Health Ministry.

**Table 3:** Variants of Concern: Mutations and their key features.

Pango Lineage/First Detected/ WHO Classification	Spike Protein Substitution	Other Mutations	Key Features
B.1.1.7 United Kingdom ALPHA (WHO)	Receptor-binding domain: N501Y Furin cleavage site: H69del, V70del, P681H Other mutations including N-terminal: A570D, T716I, S982A, D1118H Antibody supersite epitope: _Y144del, +/−E484K	NON- SYNONYMOUS Open reading frame (ORF)1ab: T1001I, A1708D, and I2230T. ORF8: Q27stop, R52I, and Y73C Nucleocapsid (N) protein: D3L and S235F SYNONYMOUS ORF1ab: C913T, C5986T, C14676T, C15279T, and T16176C Membrane (M) gene: T26801C DELETIONS ORF1ab: SGF 3675–3677del	✓ Increased transmission rates by ~50% [64] ✓ Potential increased severity (hospitalization and mortality rates) [81,82], but controlled studies indicate no increased severity [76,102] ✓ Negligible risk of reinfection [103] ✓ No effect on susceptibility to monoclonal antibody therapies ✓ Minimal impact on neutralization by convalescent and vaccine sera [62,83,104,105,106,107] ✓ No evidence of elevated reinfection rates [76] ✓ The presence of E484K led to a six-fold decrease in sensitivity of this E484K mutant virus to immune sera from individuals vaccinated with vaccine and an 11- fold decrease in sensitivity to convalescent sera [106]
B.1.351 South Africa BETA (WHO)	Receptor-binding domain: K417N, E484K, N501Y. N-terminal domain: L18F, D80A, D215G, R246I, D614G deletion at 242–244. S2 subunit: A701V.	NON- SYNONYMOUS ORF1ab: _T265I, _K1655N, H2799Y, S2900L, K3353R, D4527Y, T5912I ORF3a: Q57H, S71L Envelope (E): _P71L N viral protein: _T205I	✓ Increased transmission rates by ~50% [82] ✓ Possible increased risk of in-hospital mortality [108,109] ✓ Reduced efficacy of some monoclonal antibodies [81] ✓ Reduced neutralization by convalescent and vaccine sera [86]
P.1 Brazil GAMMA (WHO)	Receptor-binding domain: K417N, E484K, N501Y N-terminal domain: L18F, T20N, P26S, D138Y, R190S, Carboxy(C)-terminal region of the S1 domain: D614G S1 and S2 site: _H655Y, T1027I, and V1176F	NON- SYNONYMOUS Open reading frame (ORF)1ab: S1188L, K1795Q, and E5665D ORF8: E92K Nucleocapsid (N) protein: P80K SYNONYMOUS ORF1ab: C913T, C5986T, C14676T, C15279T, and T16176C Membrane (M) gene: T26801C DELETIONS ORF1ab: _SGF 3675–3677del	✓ 2.6 times more transmissible [110] ✓ Possible increased risk of hospitalization [111] ✓ Reduced efficacy of some monoclonal antibodies [112] ✓ Reduced neutralization by convalescent and vaccine sera [86,113]
B.1.617.2 DELTA (WHO)	Receptor-binding domain: L452R, T478K Carboxy(C)-terminal region of the S1 domain: D614G Furin cleavage site: P681R	Spike protein: D950N, G142D, T19R, 156del, 157del, R185G	✓ Increased transmissibility [113] ✓ Secondary household attack rate elevated [96] ✓ Possible increased risk of hospitalization [114] ✓ Potentially reduced neutralization by monoclonal antibody treatments [115] ✓ Potentially reduced neutralization by vaccine sera [116]

## 6. SARS-CoV-2 Variants of Interest

The following section references variants reported as VOI by the CDC. Mutation information is summarized in Table 4. 

## 7. Vaccines against SARS-CoV-2 Variants of Concern

### 7.1. Vaccine-Induced Immune Responses against SARS-CoV-2 Variants of Concern

During the initial stages of the COVID-19 pandemic, the number of variant viruses was small due to a smaller number of people being infected with the virus. As the infection spread worldwide, there has been an increase in the number of multiple SARS-CoV-2 variants. Controlling the spread of SARS-CoV-2 by implementing long-lasting lockdowns is not feasible due to major economic and social disruption. Hence, global public health measures, along with widespread vaccination, are the most feasible approach to contain the SARS-CoV-2 infection [117]. As discussed earlier, only four VOCs continue to be of major concern as they increase the infection rate, modify the potency of neutralizing antibodies, and thereby compromise vaccine efficacy. An effective COVID-19 vaccine will likely require both neutralizing antibodies and a Th1-driven cellular component. Here, we discuss the effect of variants of concern in terms of the immune responses elicited by the four most common vaccines and their efficacy. 

#### 7.1.1. Humoral Antibody Responses

Humoral response characterized by the presence of adequate titers of neutralizing antibody (NAb) levels is considered as an indicator of protection against SARS-CoV-2 infection [118]. The kinetics of immune response include the generation of SARS-CoV-2 specific IgM in a few days after infection, followed by the production of virus-specific IgG. 

The mRNA-based vaccine BNT162b2 (Pfizer/BioNTech vaccine) is a nucleoside-modified mRNA formulated in lipid nanoparticles that encodes the SARS-CoV-2 S stabilized in its prefusion conformation. The BNT162b2 vaccine showed strong antibody responses and poly-specific cellular immunity. Specifically, the geometric mean of the 50% neutralization titers of SARS-CoV-2 serum was up to 3.3-fold higher than that observed in samples from individuals who recovered from COVID-19 [119]. Similar results were noted with the mRNA-1273 vaccine, a lipid nanoparticle-encapsulated mRNA-based vaccine that encodes the prefusion stabilized full-length S protein of the SARS-CoV-2 developed by Moderna and the Vaccine Research Center at the National Institute of Allergy and Infectious Diseases (NIAID). The mRNA-1273 vaccine showed that the neutralizing antibody titers following two doses were similar to those found in convalescent serum specimens, and the vaccine-induced anti-SARS-CoV-2 immune responses were present in all participants within two weeks after the first vaccination [120].

The AZD1222 (ChAdOx1 nCoV-19) consists of the replication-deficient chimpanzee adenovirus-vectored vaccine, expressing the full-length SARS-CoV-2 S protein with a tissue plasminogen activator leader sequence. Vaccination with AZD1222 showed rapid production of antibodies against the SARS-CoV-2 Spike protein that peaked by day 28 and elicited the neutralizing antibody in all participants after a booster dose [121,122]. The Ad26.COV2.S is a recombinant replication-incompetent adenovirus serotype 26 (Ad26) vector encoding a full-length and stabilized the SARS-CoV-2 Spike protein. A single dose of Ad26.COV2.S in healthy individuals triggered a strong humoral response in the majority of vaccine recipients, with the presence of S-binding and neutralizing antibodies in more than 90% of participants [123,124]. Interestingly, binding and NAbs were detected by day 57 in 100% of vaccine recipients after a single immunization [125]. 

Gam-COVID-Vac (Sputnik V) is a combined vector vaccine based on recombinant adenovirus type 26 (rAd26) and recombinant adenovirus type 5 (rAd5), both of which carry the gene for SARS-CoV-2 full-length glycoprotein S. The SARS-CoV-2 RBD-specific IgGs were noted at day 14 in 88.9% in participants after administration of rAd26-S and in 84.2% of participants with rAd5-S. After day 21, a strong humoral response with RBD-specific IgGs was detected in 100% of vaccinated participants [126]. Furthermore, heterologous rAd26 and rAd5 vaccine-induced neutralizing antibodies titers were similar to those who had recovered from COVID-19 infection. 

B.1.1.7 variant: Earlier studies showed that the neutralizing activity of sera from individuals who had the BNT162b2 vaccine was modestly reduced against the B.1.1.7 variant, compared with wild-type virus [127]. Further, the escape in the neutralizing activity in the vaccine-induced sera was noted with B.1.1.7 plus E484K mutation [106]. A significant reduction in neutralization titers in serum from BNT162b2-immunized individuals was observed with a pseudovirus that contains the complete set of B.1.1.7 mutations [128]. Conversely, more recent studies report no loss of neutralizing activity against B1.1.7 compared with wild-type virus for human convalescent sera and human immune sera elicited by the mRNA-1273 and BNT162b2 vaccine [62,106,129]. Reduced neutralization capacity by vaccine sera from the mRNA-1273 and NVX-CoV2373 (two and two-fold average) were noted for the B.1.1.7 variant using the D614G variant as the comparator [104]. In contrast, no significant effect on the neutralizing capacity of sera from humans or non-human primates who received mRNA-1273 against the B.1.1.7 variant was noted [62]. 

The neutralization activity of sera from AZD1222-vaccinated individuals was ninefold lower against the B.1.1.7 variant than against a canonical non-B.1.1.7 lineage (Victoria). However, the clinical efficacy against symptomatic disease for the B.1.1.7 variant was 70.4%, indicating that either that lower neutralizing antibody titers are sufficient to provide protection or that other mechanisms of immunity could be responsible for protection from disease in vaccinated individuals [107]. Vaccination-induced antibodies cross-neutralize the variants B.1.1.7 and B.1.351, but studies indicate reduced neutralizing antibody titers against B.1.351 after vaccination but not against B.1.1.7 [82,83,130,131,132]. The Ad26.COV2-S vaccinated sera neutralized the B.1.1.7 variant in vitro, although less efficiently than the reference strain [133]. Humoral immune responses with a single dose of Ad26.COV2.S showed rapid S-specific, RBD-specific binding, and neutralizing antibody responses by day 71 [125]. 

The humoral immune response from Ad26.COV2.S-vaccinated individuals against the original SARS-CoV-2 strain, B.1.1.7, CAL.20C, P.1, and B.1.351 variants of concern showed reduced neutralizing antibody responses induced by the Ad26.COV2.S vaccine against the B.1.351 and P.1 variants [134]. Interim immunogenicity data for the Ad26.COV2.S vaccine showed humoral neutralizing antibody responses against wild type as well as B.1.1.7, B.1.617.1 (kappa), B.1.617.2, P.1, B.1.429, and B.1.351 to be similar after eight months of Ad26.COV2.S vaccine. 

B.1.351 variant: The B.1.351 variants containing the Spike K417N/T, E484K, and N501Y RBD mutations showed significantly decreased neutralization even in fully vaccinated individuals [84]. Neutralization of B.1.351 by sera from convalescent or vaccinated individuals (BNT162b2 or AZD1222) was found to be significantly reduced, and in some cases, led to a complete inability to neutralize the B.1.351 virus [132]. Neutralization by sera from individuals vaccinated with BNT162b2 showed B.1.351 to be 6.5-fold more resistant than the wild-type pseudovirus [83]. Sera from vaccinated individuals assayed for neutralization against the B.1.351 variant showed reduced neutralization activity of 10.3-fold for the BNT162b2 vaccine and 12.4-fold for the mRNA-1273 vaccine [129]. A three-fold reduction in the neutralization capacity was noted for BNT162b2 human vaccine sera against the B.1.351 spike, and this reduction in titer was attributable to the E484K mutation in the RBD [82]. A similar reduction in the neutralization activity was noted for mRNA-1273 and BNT162b2 against B.1.351 using mutant pseudoviruses [62,84,135]. Humoral antibody responses from mRNA-1273 or BNT162b2-vaccinated individuals exhibited reduced activity against SARS-CoV-2 variants containing E484K and N501Y mutations or the triple combination of K417N, E484K, and N501Y (as found in B.1.351) [85]. In another study, antibodies generated post BNT162b2 vaccination cross-neutralized B.1.1.7 and B.1.351 variants. However, the neutralizing capacity and Fc-mediated response against B.1.351 was found to be two to four-fold less than those against the wild virus [127]. Several other studies have demonstrated a significant decrease in the neutralizing capacity of sera from vaccinated or infected individuals of the South African variant [135,136]. Likewise, an analysis from Israel noted that BNT162b2 was less effective against B.1.351 than other emerging variants [137]. 

After a single dose (at week 3) of the BNT162b2 vaccine, the levels of neutralizing antibodies were low against D614G and almost undetectable against the Alpha, Beta, and Delta variants. A sixteen-fold reduction in the neutralization titers against the B.1.351 variant was noted when compared to the Alpha variant [138]. After a single dose (at week 3) of the AZD1222 vaccine, low levels of neutralizing antibodies were noted with B.1.351 variants when compared to the D614G and Alpha strains. There was, however, a nine-fold reduction in neutralization titers against the Beta variants, respectively, relative to the Alpha variant [138]. 

Gamma (P.1) variant: As the P.1 variant is shown to accumulate a very high number of S protein mutations, it is reasonable to accept that it will be similar or even more resistant than the B.1.351 variant to antibody-mediated protection. Serum neutralization assays using a pseudovirus have shown that the neutralizing activity of BNT162b2-elicited antibodies to B.1.1.7-spike virus and P.1-spike virus is approximately equivalent [136]. Similarly, the neutralizing activity of sera from BNT162b2-vaccinated individuals showed an efficient inhibitory effect with wild type and only slightly reduced effects on the B.1.1.7 variant. A markedly reduced inhibitor, the B.1.351 and P.1 variants were less susceptible to inhibition by sera/plasma from COVID-19 patients and BNT162b2-vaccinated individuals when compared to WT S protein. Hence, the markedly decreased sensitivity to antibody-mediated neutralization indicates that convalescent and vaccinated individuals might not be fully protected against infection by the B.1.351 and P.1 variants [81].

*Delta (B.1.617.2) variant.* The Delta variant has been reported to evade neutralization by antibodies induced upon infection and, perhaps more pertinent, following vaccination [138,139,140]. A significant decrease in neutralizing antibody titer has been seen for B.1.617.2 compared with B.1.1.7 using sera from individuals immunized with BNT162b2 [141]. The neutralization of B.1.617.2 using serum from individuals who had received two doses of the BNT162b2 or AZD1222 vaccine showed a reduction of 2.5-fold for the BNT162b2 vaccine and 4.3-fold for the AZD1222 vaccine. The reductions were comparable in scale with those seen with B.1.1.7 and P.1, with no evidence of widespread antibody escape as seen with the B.1.351 variant [142]. Serum-neutralizing antibodies in individuals following vaccination with two doses of ChAdOx-1 or BNT162b2 were tested on B.1.617.2 live virus isolate. The results showed a loss of sensitivity of around eight-fold for B.1.617.2 compared to Wuhan wild type for both sets of vaccine sera. Furthermore, serum neutralizing titers against B.1.617.2 were lower in ChAdOx-1 versus BNT162b2 vaccines [143].

After a single dose (at week 3) of the BNT162b2 vaccine, the levels of neutralizing antibodies were almost undetectable against the Delta variants. A three-fold reduction in the neutralization titers against the Delta variant was noted when compared to the Alpha variant. Similarly, a single dose (at week 3) of AZD1222 vaccine induced low levels of neutralizing antibodies with Delta variants when compared to the D614G and Alpha strains. There was, however, a five-fold reduction in neutralization titers against the Delta variants relative to the Alpha variant [138]. Hammerschmidt and their team studied the neutralization of the Delta variant following heterologous and homologous BNT162b2 or ChAdOx1 nCoV-19 vaccination. They found more robust humoral immune responses against the Delta variant following heterologous ChAdOx1 nCoV-19 (ChAd)/BNT162b2 (BNT) than homologous ChAd/ChAd vaccination [144]. An elevated level of intricacy in antibody responses is affected not only by the vaccine or the vaccine’s immune system but also by the VOC to be fought. 

**Table 4:** Variants of Interest: Mutations and key features.

Pango Lineage/First Detected/WHO Classification	Spike Protein Substitution	Total Mutations	Key Features
B.1.525 UK/Nigeria ETA (WHO)	A67V, del69, del70, del144, E484K, D614G, Q677H, F888L	L5F, T95I, D253G, S477N, A701V ORF1a-nsp2: T85I ORF1a-nsp4: L438P ORF1a-nsp6: 9bp deletion, delta106–108. ORF1b-nsp12: P323L ORF1b-nsp13: Q88H ORF3a: Q57H N: P199L, M234I	✓ Potential decreased neutralization by some monoclonal antibody treatment and by convalescent and vaccine sera [116]
B.1.526 New York, United States IOTA (WHO)	Receptor-binding domain: E484K, S477N Spike: L5F, T95I NTD: D253G Carboxy(C)-terminal region of the S1 domain: D614G S2 subunit: A701V.	A16500C, A22320G, T9867C	✓ Decreased susceptibility to monoclonal antibody treatments [116] ✓ Decreased neutralization by convalescent and vaccine sera [116]
B.1.617.1 India KAPPA (WHO)	T95I, G142D, E154K, L452R, E484Q, D614G, P681R, Q1071H.		✓ Potential decrease in neutralization by some monoclonal antibody treatment and by vaccine sera [142]
B.1.617.3 India	T19R, G142D, L452R, E484Q, D614G, P681R, D950N		✓ Potential decrease in neutralization by some monoclonal antibody treatment and by vaccine sera [142]

#### 7.1.2. Cellular Antibody Responses

Following infection with SARS-CoV-2, cellular responses with T cells are activated, which are crucial for containing the disease progression [145,146]. Studies indicate that specific T-cell responses are associated with milder disease in COVID-19 patients [147]. Specifically, CD4+ T cells, amongst other functions, provide signals that support the development of antibody responses. In contrast, CD8+ T cells contain the infection by eliminating already infected cells by recognizing processed antigens presented by MHC class I and II molecules. The involvement of T cells is also critical for B-cell maturation and the induction of strong and durable antibody responses [148,149]. Therefore, the generation of a robust cellular immune response is a desirable attribute for a vaccine against SARS-CoV-2. However, a potential for vaccine-associated enhanced respiratory disease (ERD) [150] due to the activation of type 2 helper T cell (Th2)-skewed cellular immune responses is a major concern [151]. Accordingly, it is deemed advantageous if a COVID-19 vaccine instead activates type 1 helper T cell (Th1)-skewed T-cell responses or balanced T-cell responses. 

The currently available vaccines for SAR-CoV-2 elicit not only neutralizing antibodies but also induce SARS-CoV-2-specific CD4+ and CD8+ T-cell responses [152]. Viral evasion from T-cell responses is difficult in comparison to neutralizing antibody response because multiple T-cell epitopes are distributed across viral proteins, whereas neutralizing antibodies targets a specific narrow region. CD8+ T-lymphocytes eliminate infected cells by recognizing viral epitopes as they are displayed on the cell surface in the context of class I major histocompatibility complex proteins (MHC-I). Some of these positions on the epitopes are important for MHC-1 presentation, and mutations in these epitope regions might interfere with peptide binding to MHC-1 [153]. With respect to SARS-CoV-2, the capacity of the virus to escape CD8+ T cell surveillance through point mutations in specific MHC-1 restricted viral epitopes (for instance, ORF8 mutations) indicates the virus to evade cell-mediated adaptive immune responses. An insignificant effect of SARS-CoV-2 variants on both CD4+ and CD8+ T-cell responses in COVID-19 convalescents and recipients of COVID-19 mRNA vaccines. The T-cell responses to the variants B.1.1.7, B.1.351, P.1, and CAL.20C were not different from those to the Wuhan strain. Despite the mutations in these variants, most of the SARS-CoV-2 T-cell epitopes were conserved [154]. Even if SARS-CoV-2 variants escape the neutralizing antibodies elicited by current COVID-19 vaccines, T-cell immunity may be helpful in reducing the disease burden of COVID-19 by attenuating disease severity and decreasing mortality.

With respect to cell-mediated immune response, BNT162b2 induced S-specific CD4+ and CD8+ T cell responses directed against RBD, S1 and S2 regions, indicating immune recognition of multiple independent MHC I and II epitopes. Interestingly, increased expression of IFNγ and IL-2 with low levels of IL-4 in BNT162b2-induced CD4+ T cells indicate a TH1 profile and the absence of a potentially deleterious TH2 immune response. Similarly, in addition, S-specific IFNγ + CD8+ T cells were also induced by BNT162b2. One study indicated that out of the 45 mutations in the B.1.351 S protein, only one mutation was associated with a low-prevalent CD8+ T cell epitope [155]. The S-specific CD4+ and CD8+ T cells induced by the BTN162b2 vaccine showed equal recognition of the wild-type, B.1.1.7, and B.1.351 S proteins [127], indicating that the B.1.1.7 and B.1.351 S proteins do not escape T-cell-mediated immunity elicited by the wild type S protein. Tarke et al. demonstrated that CD4+ and CD8+ T cell responses in convalescent COVID-19 subjects or COVID-19 mRNA vaccines (BNT162b2 and mRNA-1273) were not substantially affected by mutations found in the B.1.1.7, B.1.351, P.1, and CAL.20C variants [156]. Overall, the cumulative effect of variant mutations on the CD4+ and CD8+ T cell responses is negligible.

Nearly identical CD4+ and CD8+ T cell responses to the Delta variant peptides and SARS-CoV-2 spike peptides were detected in healthy BNT162b2-vaccinated individuals [156]. Hence, the analysis of T cell responses to VOCs (Alpha and Delta) showed that vaccination with BNT162b2 elicited equivalent T cell responses. 

The cellular immune response from Ad26.COV2.S showed Th1-biased T-cell responses (expression of IFNγ and IL-2) without TH2-cell response. CD8+ T cell responses (expression of IFNγ, IL-2, or both) were noted [123] along with IFNγ central memory T-cell responses [125]. The cellular immune response from Ad26.COV2.S-vaccinated individuals against the original SARS-CoV-2 strain, B.1.1.7, CAL.20C, P.1 and B.1.351 variants of concern showed CD8 and CD4 T cell responses, including central and effector memory responses, were similar among the Wuhan strain, B.1.1.7, B.1.351, P.1 and CAL.20C variants [134]. Interim immunogenicity data for the Ad26.COV2.S vaccine showed Spike-specific interferon-γ CD8+ and CD4+ T-cell responses to be stable over the eight-month time period [157].

The AZD1222 vaccine has been shown to induce strong Th1-skewed T cell responses to SARS-CoV-2 with CD4+ T cells primarily producing Th1 cytokines (IFNγ, IL-2, and TNFα) rather than Th2 cytokines (IL-5 and IL-13). Furthermore, IFNγ+ cells were dominant in the CD8+ T cells [122]. After the prime immunization, the S-specific T-cell responses increased as early as day 7, peaked at day 14, and were maintained up to day 56 [121]. NVX-CoV2373 vaccine-induced antigen-specific polyfunctional CD4+ T-cell responses that were reflected in high IFNγ, IL-2, and TNFα production and minimal IL-5 and IL-13 production after S protein stimulation [158].

### 7.2. Efficacy of COVID-19 Vaccines against Variants of Concern

The primary determinant conferring protection against COVID-19 is the development of antibodies against the S protein, mainly against its receptor-binding domain. This, in turn, promotes the formation of neutralizing antibodies that bind to the S protein and prevent them from infecting human cells. Many patients who have recovered from the disease exhibit high titers of SARS-CoV-2 neutralizing antibodies. Vaccines offer similar levels of protection. However, one of the most challenging considerations to develop effective vaccines against SARS-CoV-2 is the genetic instability causing a high mutation rate. To date, studies indicate that the effectiveness of the currently available vaccines may be reduced against SARS-CoV-2 variants. The various vaccines and their clinical efficacy against the VOC are described briefly and summarized in Table 5.

#### 7.2.1. Vaccine Efficacy against B.1.1.7 Variant

The Pfizer-BioNTech vaccine, also referred to as the BNT162b2 vaccine, is currently undergoing trials to determine the efficacy against the B.1.1.7 variant of concern (Table 5). An in vitro analysis demonstrated efficient neutralization of the prevalent SARS-CoV-2 variants (B.1.1.7, P.1, and B.1.351). One study measured neutralizing antibody responses after the first and second doses of the BNT162b2 vaccine using pseudoviruses modeled after the B.1.1.7 variant. Those who received the vaccine showed a broad range of neutralizing titers against the pseudovirus that were modestly reduced against this variant. This reduction was seen similarly in patients with previous COVID-19 infections. When vaccine efficacy was measured with the B.1.1.7 variant containing E484K substitution, a significant loss of neutralizing activity by the vaccine antibodies was shown versus when compared with B.1.1.7 alone. The E484K substitution could be detrimental to the efficacy of this vaccine [106]. However, the CDC reports Pfizer has displayed 90% efficacy against this variant in Qatar and 100% efficacy for severe or critical disease. This data was studied post-EUA. 

The Moderna vaccine’s, also known as mRNA-1273 vaccine, efficacy against the B.1.1.7 variant of concern is unknown. A clinical efficacy trial showed no effect on neutralization, with this variant having mutations affecting the RBD for individuals who have been vaccinated. However, the analysis represented decreased titers of neutralizing antibodies against the B.1.1.7 variant containing E484K substitution. 

The Janssen vaccine, also named the Ad26.COV2.S vaccine, has shown higher efficacy in the United States by a factor of 1.3 (72%) versus South Africa (57%). It is important to note that the B.1.1.7 variant is predominant in the U.S., and the B.1.351 lineage is predominant in South Africa. However, data for the efficacy of this single-dose regimen against B.1.1.7 is still being studied. The CDC reports, this vaccine has shown 74% efficacy in the U.S., 66% in Brazil, and 52% in South Africa. For all three countries, the vaccine has 73–82% efficacy for severe or critical disease. However, this data was studied before EUA. 

The ChAdOx1 nCoV-19 vaccine, not EUA or FDA approved in the U.S, is a two-dose regimen showing reduced neutralization activity against the B.1.1.7 variant versus non-B.1.1.7 variants. The clinical efficacy against B.1.1.7 was 70.4% versus non-B.1.1.7 variants showing 81.5% efficacy [107]. 

A recent study by Nasreen et al. showed that vaccine effectiveness with one dose for symptomatic infection was 83% for mRNA-1273, 66% for BNT162b2, and 64% for ChAdOx1 vaccines. Full vaccination (two doses) increased the effectiveness to 92% for mRNA-1273 and 89% for BNT162b2 [159]. Vaccine effectiveness against hospitalization or death after two doses was 94% for mRNA-1273 and 95% for B.NT162b2. A single dose of ChAdOx1 showed an effectiveness of 85% in preventing hospitalization or death.

According to the different studies (Table 5), the clinical efficacy of several vaccines against this variant seems to be intact and does not exhibit an escape phenomenon against vaccine-mediated protection.

#### 7.2.2. Vaccine Efficacy against B.1.351 Variant

The BNT162b2 vaccine represented vigorous neutralization but was reduced when compared to the original SARS-CoV-2 strain. Data is lacking and clinical trials are currently being investigated to determine efficacy against this variant [105,106]. The mRNA-1273 vaccine represented decreased titers of neutralizing antibodies for this VOC. Compared to the other current VOC reported by the CDC (B.1.1.7, P.1, B.1.427, and B.1.429), the reduced neutralizing titers were significantly lower in this particular lineage. This means the vaccine is less efficacious against B.1.351 when compared to the other prevalent concerning variants [160]. These data are supplemented by a study from Israel, which found infections in vaccinated individuals were eight times more likely to be caused by B.1.351 than in unvaccinated individuals, indicating reduced vaccine efficacy against B.1.351 [137]. Similarly, a >5% reinfection rate that did not differ between seropositive and seronegative people was noted, indicating a lack of protection in response to previous infections [161].

Moderna is currently investigating to see if a third booster dose of the current mRNA-1273 vaccine would increase the neutralizing titers against the B.1.351 and other variants. Furthermore, Moderna is investigating a booster vaccine candidate called mRNA-1273.351 against the B.1.351 variant and a bivalent vaccine with a 1:1 mix of original and variant vaccine (mRNA-1273.211) [162].

The Ad26.COV2.S vaccine offers protection across South Africa, where the predominant variant is B.1.351. However, data has not been reported yet. One study has reported 57% efficacy in South Africa [163].

Protection against symptomatic infection with one dose showed 60% effectiveness for BNT162b2, 48% for ChAdOx1 and 77% for mRNA-1273. Vaccine effectiveness against hospitalization or death after two doses was 95% for B.NT162b2. A single dose of mRNA-1273 and ChAdOx1 showed the effectiveness of 89% and 83% in preventing hospitalization or death [159].

A multicenter, double-blind, randomized control trial utilizing the ChAdOx1 nCoV-19 vaccine did not show efficacy towards mild to moderate infection with the B.1.351 variant in South Africa [130]. Minimal data is available and clinical efficacy is still being studied.

#### 7.2.3. Vaccine Efficacy against the P.1 Variant

Protection against symptomatic infection with one dose showed 60% effectiveness for BNT162b2, 48% for ChAdOx1, and 77% for mRNA-1273. Vaccine effectiveness against hospitalization or death after two doses was 95% for B.NT162b2. A single dose of mRNA-1273 and ChAdOx1 showed an effectiveness of 89% and 83% in preventing hospitalization or death [159].

The BNT162b2 vaccine has shown similar neutralization patterns against both B.1.1.7 and the P.1 variants. Data is still needed, but neutralization has been demonstrated with the vaccine-elicited antibodies against this variant of concern. The mRNA-1273 vaccine has shown decreased neutralizing antibody titers against the P.1 variant. However, the efficacy of this vaccine against the VOC is still unknown.

#### 7.2.4. Vaccine Efficacy against the B.1.617.2 Variant

A study published in the Lancet found that the two-dose regimen of Pfizer BNT162b2 provided 79% protection from the Delta variant compared to 92% protection against the Alpha variant. With respect to the Oxford-AstraZeneca vaccine (ChAdOx1 nCoV-19), the protective effect was substantially reduced from 73% for the Alpha variant versus 60% for the Delta variant [140]. Similarly, BNT162b2 two-dose effectiveness reduced from 93.4% for the Alpha variant to 87.9% for the Delta variant. With the ChAdOx1 vaccine, the effectiveness decreased from 66.1% for the Alpha variant to 59.8% for the Delta variant [139]. Nevertheless, vaccine efficacy against preventing hospitalization for the Delta variant was 96% after two doses of BNT162b2 and 92% after two doses of ChAdOx1 [164]. The numbers of cases and follow-up periods are currently insufficient to estimate effectiveness against severe disease, including hospitalization and mortality.

Vaccine effectiveness after a single dose was 56% for BNT162b2, 72% for mRNA-1273, and 67% for ChAdOx1. Full vaccination with BNT162b2 provided 87% protection against Delta. Vaccine effectiveness against hospitalization or death after single dose was 78% for B.NT162b2, 96% for mRNA-1273, and 88% for ChAdOx1 vaccine [159]. More data is required to determine vaccine efficacy against the Delta variant.

**Table 5:** Vaccines Efficacy and effectiveness against Variants of Concern.

Vaccine Name	Non-Variant and Variants of Concern					
		Non-Variant	B.1.1.7 (Alpha)	B.1.351 (Beta)	P.1 (Gamma)	B.1.617.2 (Delta)
**Pfizer** **Comirnaty (BNT162b2)**	Overall efficacy:	95% [165,166]	90–93% 90% [167] Similar to non-variant [118,121]	75% [165]	No evidence of reduced protection	75–82% [139] Single dose: ~88% [168]
	Asymptomatic disease:	N/A	Israel: 90.7–92.2% [159]	N/A	N/A	N/A
	Symptomatic disease:	Trial: 89–93.2% Israel CLALIT: 87–98% [169] England (80 + years): 85–93% [170]	Israel: 96.7–97.2% Canada: 89% [159]	Canada: 84% two doses [159]	Canada: 84% two doses [159]	Single dose: 33% Two doses: 88% [139] ~88% [164] Canada: 87% [159]
	Severe disease:	UK Siren: 95.3–100% [171] Israel CLALIT:75–100% [169] England (80 + years): 37–62% lower risk of mortality 80 + years [172] Israel (85 + years): 97% 7 days after 2nd-dose decreased mortality [173]	Qatar: 81.7–100% [165]	Qatar: 92.2–99.5% [165]		N/A
	Reduced hospitalization:	England (80 + years): 33–52% in 80 + years [172] Israel (85 + years): 96.9% in 85 + years [173]	Canada: 95% [159]	Canada: 95% (two doses) [159]	Canada: 95% (two doses) [159]	Single dose: 46–99% Two doses: 86–99% [164] Scotland: 92% [140] Canada: 78% (single dose) [159]
	Decreased infection:	UK Siren: 76–97% [171] Israel CLALIT: 88–95% [169] Israel: 94.1% in 85 + years [173]	Qatar: 85.9–92.3% [165]	Qatar: 70.5–78.9% [165]		N/A
**Moderna COVID-19 vaccine** **(mRNA 1273)**	Overall efficacy:	94.1% [174] 89.3–96.8% [174] >90% [175] >65 years: 61.4–95.2% Adolescents: 100% [176]	N/A	6.5-fold reduction in neutralizing antibodies in laboratory study [160]	2.6-fold reduction in neutralizing antibodies in laboratory study [160]	
	Asymptomatic disease:	Single dose: 80% after 14 days Two doses: 90% after 14 days [125]	N/A	N/A	N/A	N/A
	Symptomatic disease:	89.3–96.8% (Trial) Single dose: 80% after 14 days Two doses: 90% after 14 days [177]	Canada: 92% [159]	Canada: One dose 77% [159]	Canada: One dose 77% [159]	Canada: One dose 72% [159]
	Severe disease:	100% [174] >95% [175]	N/A	N/A	N/A	N/A
	Reduced hospitalization:	N/A	Canada: 94% (two doses) [159]	Canada: 89% (single dose) [159]	Canada: 89% (single dose) [159]	Canada: 96% (single dose) [159]
**AstraZeneca/Oxford** **COVID-19 Vaccine** **AstraZeneca** **(AZD1222);**	Overall efficacy:	55–81% [178] 58–95% in 65 + years (US Study) 27–90% after 35 + days in 70 + years [172] 60–81% in 80 + years [170]	~75% [107,178]	≤10% [130]	Antibody titers reduced by 2.9-fold [179] 40.7–69.7% (Trial, non-variant specific)	53–66% [140] Single dose: 60–71% [164]
	Symptomatic disease:	70.7–91.4% [107] 68–82% [180] 55–81% [178]	43.6–84.5% [107,181] Two doses: 66% [172]	-76.8–54.8% [130] Canada: One dose 48% [159]	Canada: One dose 48% [159]	Single dose: 33% Two doses: 60% [139] ~67% [164] Canada: One dose 67% [159]
	Severe disease:	100% [180]	N/A			N/A
	Reduced hospitalization:	100% [180] 60–81% in >80years [170] 74–89% in 70–79 years [170]	Canada: Single dose 85% prevention [159]	Canada: Single dose 83% prevention [159]	Canada: Single dose 83% prevention [159]	Single dose: 51–83% Two doses: 75–97% [164] 92% [182] Scotland: 73 [140] Canada: 88% (single dose) [159]
	Decreased infection:	42–60.1% [107] Single dose: 60–70% [183]	N/A	N/A	N/A	N/A
**Johnson and Johnson** Ad26. CoV2-S	Overall efficacy:	66% [184]	30.3–95.3% [185] 70% [133]	72% (USA) 66% (Latin America) 57% (South Africa) [133]	68% [133]	60% [185]
	Symptomatic disease:	58.2–81.7% [133] Moderate-severe: 59–73.4% [133]	N/A	41.2–78.7% [132]	48.8–80.7% [132]	
	Severe disease:	9.4–99.7% [133] Severe-critical: 54.6–89.1% [133] 85.4% [184]	N/A	46.2–95.4% [133]	7.8–99.7% [133]	N/A
**Novavax** NVX-CoV2373	Overall efficacy:	89% [186]	~85.6% [187] ~86% [187,188]	~49% [186] 60% [187]	No evidence yet	No evidence yet
	Symptomatic disease:	73.8–99.5% [188] 100% against severe disease and hospitalization [187,189]	71.3–93.5% [188]	28.4–63.1% [133] 6.1–72.8% [137] 60% [137]		
**Sputnik V (also known as Gam**- **Covid-Vac)**	Overall efficacy:	~91% [137]	No evidence yet			No evidence yet
	Symptomatic disease:	85.6–95.2% [190]		No evidence yet	No evidence yet	
	Severe disease:	94.4–100% [191]				

## 8. Countermeasures to Treat Anticipated Continued Viral Mutations

The emergence of several new variants has raised the question of the efficacy of the currently available vaccines against SARS-CoV-2. The efficacy of various vaccines against the VOC has been studied under multiple clinical settings, although they are still preliminary. Further studies with large sample sizes across the globe are required to better understand the newly emerging mutants. Some of the additional measures which could be implemented are as follows. 

Increasing evidence indicates that certain vaccines are less efficacious in offering complete protection against the variants. Periodically updating the approved vaccines concerning clinical efficacy against the variants are required as most of the efficacy data obtained so far are based on infection by the original strain. For instance, the efficacy of the NVX-CoV2373 vaccine against variants has been studied in phase III clinical trial, and the results indicate that the efficacies of the NVX-CoV2373 vaccine against the original COVID-19, the U.K. variant, and the South African variant strains were 95.6%, 85.6%, and 49.4%, respectively [191]. 

The currently available vaccines have shown great efficacy in preventing severe COVID-19 disease; however, it is unclear how long the immune protection from vaccination will last. As new variants emerge and continue to spread and mutate, immune response due to vaccination becomes less effective. Another growing concern is the rise in vaccine breakthrough cases [192]. Hence, additional booster doses after receiving the conventional first two vaccinations (“prime-boost” course) might be necessary for the general population as well as the high-risk individuals after a period of time to provide the required protection. A new clinical trial named COV-Boost is underway to assess the effect of a third “booster” dose of seven different Covid-19 vaccines (Oxford-AstraZeneca, Pfizer-BioNTech, Moderna, Novavax, Valneva, Janssen, and Curevac) on patients’ immune responses in the United Kingdom [193]. More studies are underway to determine when the current vaccine’s protection begins to decline. The second strategy would be to investigate the alternating vaccine immunization strategy to improve the immunogenicity of the vaccines. The first clinical trial to investigate four different combinations of prime and booster dosing by alternating vaccines (Oxford-AstraZeneca and Pfizer vaccines) is underway at The University of Oxford [194]. Similarly, a combined clinical trial using the Sputnik V and AstraZeneca vaccines is being considered [195]. A further consideration is being taken by the US Food and Drug administration in mixing and matching vaccines [196]. Finally, utilizing multivalent vaccines could be effective in preventing the spread of the variants. For instance, GlaxoSmithKline plc and CureVac N.V. are developing next-generation mRNA multivalent vaccines [197].

Considering that the vaccination coverage globally is less than expected, extending the dosing schedules between first and second doses from 4 weeks to 12 weeks has been implemented in order to improve the number of protected individuals and reduce the risk of transmission. However, one of the limitations of this approach is that significant proportions of individuals, especially the elderly age group, exhibit low titers of neutralizing antibodies for the additional eight weeks [198]. Hence, there is an increased risk of viral transmission in those individuals with low neutralizing antibodies during these extended dosing schedules.

Post-vaccination, T cell-mediated immunity has been shown to confer protection against COVID-19, especially against severe disease [199]. As the emerging variants have been shown to exhibit increased resistance to antibodies, studies need to be conducted to confirm if the T cell immune responses are also affected or if the protective effect of T lymphocytes against infection is altered. Furthermore, strategies to protect COVID-19 infection in immunocompromised individuals who have impaired T cell immune response and are, therefore, unable to mount responses even after vaccination, need to be evaluated.

The design and development of vaccines against the emerging variants need to be considered. During the pre-clinical studies, the development of a transgenic or humanized animal model for SARS-CoV-2 variant infection could aid in vaccine and drug development. For instance, Hansen et al. developed a humanized mouse model and compared the similarities and consistencies of antibodies against the SARS-CoV-2 S protein produced by humanized mice and convalescent patients [200]. Monoclonal antibodies that are authorized for emergency use are effective only against the initial variant. Since the new variants could reduce the effectiveness of neutralizing antibodies, these monoclonal antibodies should be tested against the emerging variants to confirm the efficacy. Additionally, a combination of antibodies targeting other viral regions along with the S protein could help in combating the escape variants. Lastly, approaches such as “lethal mutagenesis” targeting the viral RNA-dependent-RNA-polymerase (RdRp) could also prove to be beneficial [201]. 

To decrease the viral transmission and the frequency of the mutations, abiding by public health control measures, such as wearing masks, social distancing, and personal hygiene, are essential. Since the new variants have higher transmissibility, formulating and following public health control measures is one of the most effective and economical strategies to deal with the crisis. To prevent or overcome the vaccine escape, modifying the vaccination regimen, for instance, the addition of a booster dose, would be ideal. Furthermore, strategies such as combining different vaccine platforms for the new variants would be beneficial. Finally, intense monitoring and data collection are employed to ensure the best vaccine strategy to combat this pandemic. 

## 9. Conclusions

The current COVID-19 epidemic has an unpredictably uncertain future. Constant monitoring for the emergence of variants of concern, as well as detailed research of their influence on public health, will be required. Vaccine development has become a high priority for global health due to the destructive impact of the ongoing COVID-19 epidemic on public health, the economy, and society. Although these new variants of SARS-CoV-2 escape recognition by vaccine-induced immunity, T-cell immunity may have an essential role in reducing the disease burden of COVID-19 by decreasing the severity of the disease and mortality. Further research and investigation need to be performed to assess the emerging and evolving variants of concern, the effectiveness of the vaccines, and decrease the rate of transmission, preventing hospitalization, complications, and death. Most of all, public health policies should be executed to safeguard the concerns about SARS-CoV-2 variants, and potential lowered vaccine efficacy involving variants of concern does not result in reduced COVID-19 vaccination rates. 

## Figures and Tables

**Table 1:** Classification of SARS-CoV-2 Variants.

	Pango Lineage and Corresponding WHO Naming	Attributes *
Variants of Interest	• B.1.525 (Eta) • B.1.526 (Iota) • B.1.617.1 (Kappa) • B.1.617.3 • C.37 (Lambda) ^ • B.1.621 (Mu) ^#^	• Changes to receptor binding • Reduced neutralization by antibodies generated from previous infection or vaccination • Reduced efficacy of treatments • Potential diagnostic impact • Predicted increase in transmission and disease severity • Limited prevalence or expansion in the U.S. or other countries
Variants of concern	• B.1.1.7 (Alpha) • B.1.351 (Beta) • P.1 (Gamma) • B.1.617.2 (Delta)	• Attributes of variants of interest, but also includes: • Increase in transmissibility • More severe disease • Significant reduction in neutralization by antibodies generated during previous infection or vaccination • Reduced effectiveness of treatments or vaccines • Diagnostic detection failures and widespread interference with diagnostic test targets • Evidence of reduced vaccine-induced protection from severe disease
Variants of high consequences	• None (as of 17th September 2021)	• Attributes of variants of concern but also includes: • Demonstrated failure of diagnostics • Significant reduction in vaccine effectiveness, a high number of vaccine breakthrough cases, and very low vaccine-induced protection against severe disease • Significantly reduced susceptibility to multiple Emergency Use Authorization (EUA) or approved therapeutics • More severe clinical disease and increased hospitalizations

The classification is as per the Centers for Disease Control and Prevention (CDC) dated 17th September 2021. * As per the CDC definition. ^ The World Health Organization (WHO) named it the Lambda variant on 14th June 2021. ^#^ The World Health Organization (WHO) included the Mu variant as a variant of interest on 30th August 2021.

**Table 2:** Predominant Spike Mutations associated with SARS-CoV-2 variants.

Name of the Mutation	Location in Spike Protein	Important Effects
N501Y	Receptor-Binding Domain	✓ Increased interaction with ACE2 receptor ✓ Increased binding affinity ✓ Escapes some neutralizing antibodies ✓ K417N attenuates affinity for ACE2 but compensated by N501Y mutation [49]
K417N and K417T	Receptor-Binding Domain	✓ Conformational change in the S protein [50,51] ✓ Escapes some neutralizing antibodies ✓ K417N attenuates affinity for ACE2 but compensated by N501Y mutation [49]
E484K, E484Q, and E484P	Receptor-Binding Domain	✓ Immune escape ✓ Loss of monoclonal antibody efficacy [52] ✓ Decreased sera neutralization [53]
L452R	Receptor-Binding Domain	✓ Increased transmissibility [54] ✓ Immune escape ✓ Loss of monoclonal antibody efficacy [52] ✓ Decreased sera neutralization [53]
S477G, S44N, and S477R	Receptor-Binding Domain	✓ Increased affinity for ACE2 receptor ✓ Result in escape from some monoclonal antibodies [55]
R246I/M	N-terminal domain (adjacent to the glycosylation sites)	✓ May aid in neutralizing antibody resistance [27]
69del and 70del	N-terminal domain	✓ Alter the conformation of an exposed NTD loop and thereby increase in infectivity [56] ✓ Decreased sera neutralization [57] ✓ S-gene target failure (SGTF) in multiplex RT-PCR tests [58]
S13I and W152C	N-terminal domain	✓ Escape from monoclonal antibodies against the N-terminus [59]
L18F	N-terminal domain	✓ Immune escape from neutralizing antibodies against the N-terminus [5]
141–143del	N-terminal domain	✓ Monoclonal Antibody escape [57]
144del	N-terminal domain	✓ Resistance to 4A8 monoclonal antibody [57]
D614G	Carboxy(C)-terminal region of the S1 domain	✓ Increased binding for ACE2 receptor [60] ✓ Modest infectivity increase
H655Y	Adjacent to the S1/S2 cleavage site	✓ mAb escape, linked to feline propagation [61]
P681H/R	Adjacent to the S1/S2 cleavage site	✓ May affect transmissibility ✓ Possible increased infection
